# Chinese herb cinobufagin-reduced cancer pain is associated with increased peripheral opioids by invaded CD3/4/8 lymphocytes

**DOI:** 10.18632/oncotarget.14005

**Published:** 2016-12-17

**Authors:** Tao Chen, Shenjun Yuan, Xin-nian Wan, Ling Zhan, Xue-qin Yu, Jian-hong Zeng, Hong Li, Wen Zhang, Xiang-yang Hu, Yi-fei Ye, Wei Hu

**Affiliations:** ^1^ Third-Grade Pharmacological Laboratory on Chinese Medicine Approved by State Administration of Traditional Chinese Medicine, China Three Gorges University, Yichang 443002, China; ^2^ Department of Pharmacology, China Three Gorges University, Yichang 443002, China; ^3^ College of Medical Science, China Three Gorges University, Yichang 443002, China; ^4^ Hubei Key Laboratory of Tumor Microenvironment and Immunotherapy, China Three Gorges University, Yichang 443002, China

**Keywords:** cinobufagin, cancer pain, β-endorphin, peripheral opioid receptor, lymphocytes

## Abstract

**Objectives:**

To investigate the mechanism of cinobufagin-reduced cancer pain in mouse cancer pain model and *in vitro* cell co-culture system.

**Methods:**

Female Kunming mice were randomly divided into 4 groups. One group of animals was set as normal control without any treatment. Other three groups of animals received H22 hepatoma cell inoculation in right hind paw. At day 9 after inoculation, mice in other three groups were injected intraperitoneally once a day for 8 days with the solvent, morphine or cinobufagin, respectively. The pain behavior was recorded daily. On the last day, all mice were sacrificed and xenograft tissues homogenate and plasma levels of β-endorphin (β-END), corticotropin-releasing factor (CRF) and interleukin-1β (IL-1β) were assessed by ELISA assay. Immunohistochemistry was performed to determine the expression of β-END, pro-opiomelanocortin (POMC) and the μ-opioid receptor (μ-OR) in the xenograft tissues. Immunofluorescence was used to localize lymphocytes with expression of CD3^+^, CD4^+^ and CD8^+^ in xenograft tumors and adjacent tissues. Mice splenic lymphocytes and H22 hepatoma carcinoma ascites cells were prepared for co-culture. β-END and CRF were detected in co-culture supernatants. The MTT assay and cytometry were used to assess cell proliferation. RT-PCR was conducted to determine the gene expression of POMC and Cathepsin L (CTSL). Chemotaxis was examined using a transwell-based migration assay.

**Results:**

Compared to the model group, the thermal and mechanical pain thresholds were increased in mice after cinobufagin treatment. The expression of β-END and CRF in the plasma and tumor tissues of cinobufagin group were much higher than that of the model group mice, but the expression of IL-1β in the plasma and tumor tissues was much lower than that in the model group mice. Meanwhile, the expression of β-END, POMC and μ-OR proteins was significantly increased in the xenograft tissues from cinobufagin group. Lymphocyte population of CD3^+^, CD4^+^, CD8^+^ were also elevated in xenograft tumors and adjacent tissues. In the cell co-culture assays, the content of β-END in the supernatant was significantly increased by cinobufagin in a dose-dependent manner. Cinobufagin also largely increased the proliferation of immune cells and inhibited H22 hepatoma carcinoma cell proliferation in single or co-culture cell assays. Gene expression of POMC and CTSL in cinobufagin group was significantly up-regulated comparing to the control group. Finally, cinobufagin addition enhanced the migration of immune cells in transwell assay.

**Conclusions:**

Cinobufagin-induced local analgesic effect might be associated with increased activity of POMC/β-END/μ-OR pathway released from invaded CD^3/4/8^ lymphocytes in cancer tissues.

## INTRODUCTION

Cancer pain is a complicated syndrome in cancer patient via distinct causes from cancer mass itself or by its treatment such as surgery, chemotherapy, radiotherapy. Cancer pain often times leads to mental, psychological and even ‘social pain’. It produces anxiety, depression and negative feelings of worth. Unrelieved cancer pain is associated with high levels of depression and anxiety, and can greatly interfere with daily functioning, including general activity, mobility, relationships with others, sleep, and enjoyment of life [1]. Although there are a variety of methods applying to treat cancer pain, such as bisphosphonates, chemotherapy, surgery, nerve block, adoptive tumor immunotherapy, and gene knockout, the clinic treatment of cancer pain is still to focus on the three-step program. However, many patients tortured by cancer pain could still not been controlled appropriately, and there are many problems needed to be solved now, such as “mirror pain”, morphine tolerance, constipation, respiratory depression for opioid drugs, and stomach ulcers and kidney toxicity for nonsteroidal anti-inflammatory analgesics. The clinical use of these drugs could be limited by these side effects [2].

Although pain has been extensively studied, patient's cancer pain is still not well controlled. Poorly controlled pain is a significant problem for cancer patients. Contributing factors may include concerns about analgesics and fears about the implications of pain, which may hinder open communication. It has been repeatedly demonstrated that opioid is one of the most effective drugs for cancer pain currently [3–4]. Opioid drugs combined with opioid receptors to produce their analgesic effect after treatment in patients, and more than 80% of patients with cancer need to use opioids to improve or control pain. However, the accompanying side effects of opioid drugs such as tolerance, addiction, excitement, drowsiness, constipation, nausea, vomiting, and respiratory depression limit the further application [5–7]. Systemic and central administration of opioids for the treatment of pain is sometimes accompanied by serious adverse side effects, and tolerance often occurs with prolonged use. The targeting of peripheral opioid receptors may provide pain relief. The peripheral analgesic effects of opioids have been demonstrated in a variety of animal models; however, clinical results of peripheral opioid therapy remain inconsistent [8]. Further study is needed to develop these agents into clinical therapy.

Pro-opiomelanocortin (POMC) is mainly expressed in corticotropic cells of the anterior pituitary lobe and in melanotropin cells of the intermediate lobe but immune cells involved in local inflammation also express POMC, which enzymatically by Cathespin L (CTSL) to give rise to a series of peptide hormones including α-MSH, adrenocorticotropic hormone (ACTH) and β-endorphin (β-END), etc [9]. Cytokines, including corticotropin-releasing factor (CRF) and interleukin-1 (IL-1), combine with receptors on immune cells and stimulate β-END release into the extracellular. β-END acts on opioid receptors, on nerve endings involved in local inflammation tissue, to produce endogenous analgesia.

It has been shown that activation of immune cells is positively correlated with their content of opioid peptides, with activated lymphocytes exhibit a high expression of opioid peptides [10]. The locally released opioid peptides combine with opioid receptors to produce analgesia, indicating that the number of immune cells plays a key role in the peripheral analgesic process. In injured tissues, inflammatory chemokines stimulate immune cells to increase their membrane permeability and migrate to the injured tissues. The process is mediated by various adhesion molecules including L-selectin, P-selectin on endothelial cells and E-selectin [11–12], which are located in immune cells and the vascular endothelium. Opioid peptides released by immune cells and the reactions of an aggregation of immune cells are also correlated with inflammatory chemokines [13–14].

Chinese medicine has long been shown to have efficacy in treating cancer pain, producing an elevated pain threshold, reducing the body's response to the cancer and changing the spiritual environment of the patient. It also exerts long effective time with fewer side effects [15–16]. Cinobufagin injection is a water-soluble extract from the skin of the toad *Bufo bufo gargarizans* Cantor and is effective on a variety of cancer pain [17]. Especially, when combined with chemotherapy drugs, it not only reduces the pain in a large extent, but also reduces the side effects of chemotherapy drugs with improved the overall quality life of patients [18]. The analgesic effect of cinobufagin could be blocked by a selective peripheral opioid receptor antagonist naloxone tetravalent salt derivative (NAL-M) that cannot pass the blood-brain barrier, indicating that the analgesic effect of cinobufagin is mediated by a mechanism through peripheral rather than central opioid receptors [19–20].

We recently reported [20] that cinobufagin injection treatment increased the thresholds of thermal pain and mechanical pain, which was blocked by the peripheral opioid receptor antagonist NAL-M. In parallel, β-END, POMC and μ-OR expression was increased in animals after cinobufagin injection treatment. In this study, we further dissected the mechanism for cinobufagin-reduced cancer pain by exploring the involvement of lymphocytes in releasing β-END in tumor tissue. Our data revealed that in cinobufagin-treated animals, tumor infiltrating lymphocytes (CD3^+^, CD8^+^, CD4^+^) levels were largely increased in xenograft tumors, indicating intra-tumoral inflammation might play a role in cinobufagin-induced cancer pain release.

## RESULTS

### Effect of cinobufagin on thermal hyperalgesia and mechanical hyperalgesia

**Figure 1:**
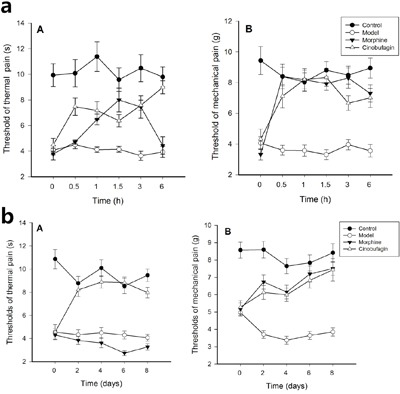
Changes of thermal hyperalgesia and mechamical hyperalgesia thresholds Data are shown as the mean ± SD (*n* = 12). (A) Thermal hyperalgesia threshold, (B) Mechamical hyperalgesia threshold. **a**. Thermal hyperalgesia and mechamical hyperalgesia thresholds at different time after the first administration of cinobufagin. The pain behavior was recorded before treatment and at 0.5, 1.0, 1.5, 3 and 6h after initial administration. **b**. Thermal hyperalgesia and mechanical hyperalgesia thresholds, at different time after administration of cinobufagin. The pain behavior was recorded before treatment and on the 2nd, 4th, 6th, and 8th day after administration.

The threshold of thermal and mechanical pain of mice in the control group was much higher than that following inoculation of H22 hepatoma cells. Compared with the model group, the threshold of thermal and mechanical pain was increased 0.5, 1.0, 1.5, 3, 6 h after the initial administration of cinobufagin. In morphine treated mice, the threshold of thermal was higher from 0.5-3 h after the initial administration than model group mice (*P* < 0.01), but equal with model group mice after 6 h initial administration. The threshold of mechanical pain of cinobufagin and morphine group mice were higher than that of model group mice from the 0.5 h after initial administration (*P* < 0.01) (Figure 1a).

After continuous administration for 8 days, compared with the model group, the threshold of thermal and mechanical pain in the cinobufagin group was significantly increased 2, 4, 6, and 8 days after initial drug administration. In morphine treated mice, the threshold of thermal was lower than model group mice on the 2th day after continuous administration, while the threshold of mechanical pain of cinobufagin and morphine group mice have no obvious difference (*P* > 0.05) (Figure 1b).

### Effect of cinobufagin on cancer pain model *in vivo*

**Figure 2:**
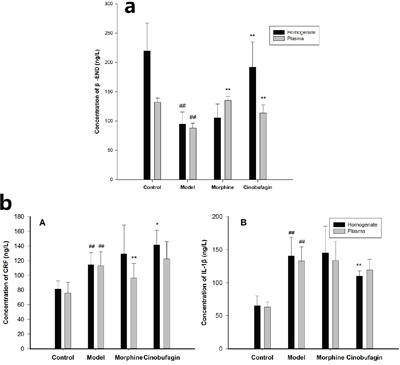
Effect of cinobufagin on the expressions of β-END, CRF and IL-1β **a**. Effect of cinobufagin on the expressions of β-END in tumor tissue homogenate and plasma. The expression of β-END in model mice was much lower than in control mice. The expression of β-END in tumor tissue homogenate and plasma in cinobufagin administration group was much higher than that of model mice. **b**. Effect of cinobufagin on the expressions of CRF and IL-1β in tumor tissue homogenate and plasma. (A) Expressions of CRF; (B) Expressions of IL-1β. The expression of CRF and IL-1β in tumor tissue homogenate and plasma in the cancer pain model mice was higher than that of control mice. The expression of CRF in tumor tissue homogenate and plasma in cinobufagin administration group was much higher than that of model mice, but IL-1β in tumor tissue homogenate and plasma in cinobufagin administration group was much lower than that of model mice. Compared with control group, ^#^*P* < 0.05, ^##^*P* < 0.01; compared with model group, *P < 0.05, **P < 0.01.

The expression of β-END in plasma and tumor tissues homogenate in model group mice was much lower than that of control group mice (*P* < 0.01). In contrast, cinobufagin significantly enhanced the expression of β-END in the plasma and tumor tissues homogenate compared with the model group mice (*P* < 0.01) (Figure 2a). The expressions of CRF and IL-1β in the plasma and tumor tissues homogenate in model group mice was much higher than that in the control group mice (*P* < 0.01). After treatment with cinobufagin, cinobufagin could up-regulate the expression of CRF and down-regulate the expression of IL-1β in plasma and tumor tissues homogenate than the model group mice (Figure 2b).

**Figure 3:**
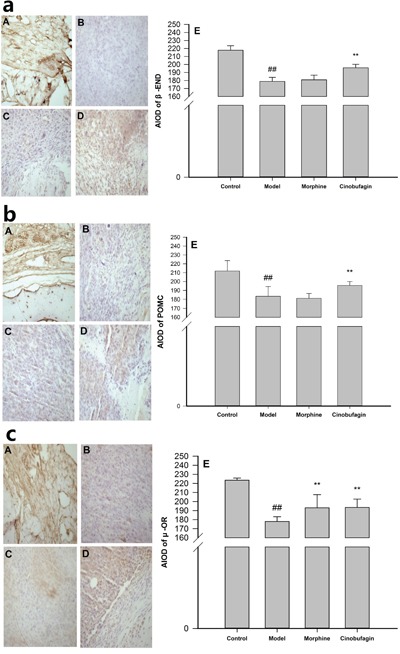
Effect of cinobufagin on the protein expressions of β-END, POMC and μ-OR (A) Control group, (B) Model group, (C) Morphine group, (D) Cinobufagin intraperitoneal group, (E) Analysis of the area of integral optical density (AIOD). **a**. Effect of cinobufagin on the protein expressions of β-END (400×). There was obvious expression of β-END and a widespread distribution of positive brown-staining in many cell membrane in cinobufagin group mice. **b**. Effect of cinobufagin on the protein expression of POMC (400×). There was obvious expression of POMC and widespread distribution of positive brown-staining was observed in many cell membrane in cinobufagin group mice. **c**. Effect of cinobufagin on the protein expression of μ-OR (400×). There was obvious expression of μ-OR and widespread distribution of positive brown-staining in many cell membrane in cinobufagin group mice. Compared with control group, ^#^*P* < 0.05, ^##^*P* < 0.01; compared with model group, *P < 0.05, **P < 0.01.

The immunohistochemistry results unequivocally demonstrated that in the control group mice, there was an obvious expression of β-END, POMC and μ-OR, and widespread distribution of positive brown-staining in many cell membranes. However, there were few positive colored areas and even fewer buff-colored cells in the model group mice compared with the control group mice, the expression of β-END, POMC and μ-OR protein in the tumor tissues were significantly reduced (*P* < 0.01) (Figure 3). Cinobufagin markedly increased the number of cells that expressed β-END, POMC and μ-OR, and the number of positive-colored areas was much higher than in model group mice (*P* < 0.01), while morphine had little influence on the expression of β-END and POMC (Figure 3a, 3b) and up-regulate the expression of μ-OR (*P* < 0.01) (Figure 3c).

**Figure 4:**
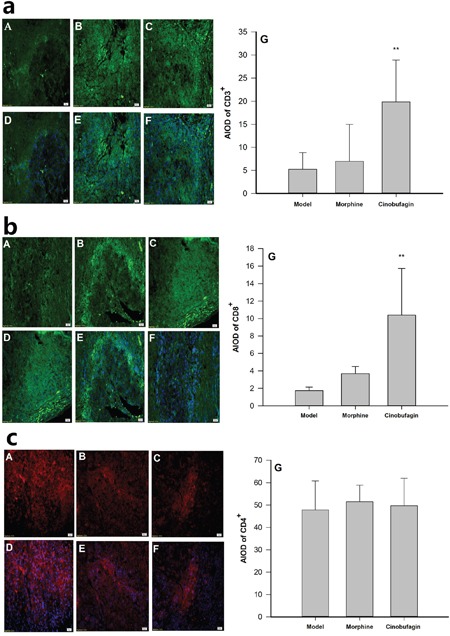
Effect of cinobufagin on the protein expression of CD3^+^, CD8^+^ and CD4^+^ (A, D) Model group; (B, E) Morphine group; (C, F) Cinobufagin group; (G) Analysis of the area of integral optical density (AIOD). A, B and C were protein staining; D, E and F were nuclear staining. **a**. Effect of cinobufagin on the protein expression of CD3^+^ (400×). The expression of CD3^+^ was observed in the cell membranes. Fluorescence showed as a dark bright green. **b**. Effect of cinobufagin on the protein expression of CD8^+^ (400×). The expression of CD8^+^ was observed in cell membranes. Fluorescence showed as a dark bright green. **c**. Effect of cinobufagin on the protein expression of CD4^+^ (400×). The expression of CD4^+^ was observed in cell membranes. Fluorescence showed as a dark bright red. Compared with model group, *P < 0.05, **P < 0.01.

The immunofluorescence results clearly revealed the protein expressions of CD3^+^, CD8^+^ and CD4^+^ were observed in cell membranes as demonstrated by dark bright green or dark bright red flourescence (Figure 4). The protein expressions of CD3^+^, CD8^+^ in cinobufagin group mice were much higher than that in model group mice (*P* < 0.01) (Figure 4a, 4b), but the protein expression of CD4^+^ in cinobufagin group mice was no obvious difference compared with that of model group mice (*P > 0.05*) (Figure 4c).

### Effect of cinobufagin on the co-culture model *in vitro*

**Figure 5:**
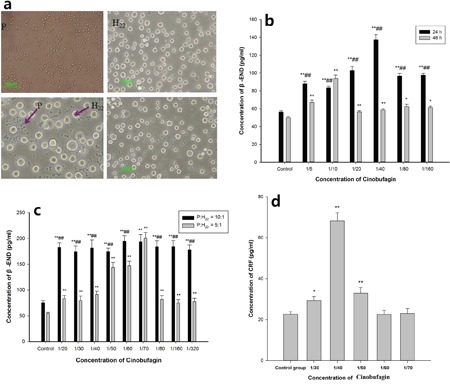
Effect of cinobufagin on the expression of β-END and CRF in the co-culture model **a**. Single cell morphology and co-culture status. (P) spleen lymphocyte, (H_22_) H_22_ hepatoma cells; spleen lymphocytes were round, small, the cell membrane was integrated and refraction was good; H_22_ hepatoma cells were round, some were large and some were small, the cell size is a multiple of spleen lymphocytes. **b**. Effect of cinobufagin on the expression of β-END at different times. After 24 h, different concentrations of cinobufagin promoted the release of β-END from immune cells, and after 48 h, different concentrations of cinobufagin still promoted its release. **c**. Effects of different concentrations of cinobufagin on the expression of β-END. When spleen lymphocytes and H22 hepatoma cell concentration ratio was 10:1 or 5:1, different concentrations of cinobufagin stimulated immune cells to release β-END. The release of β-END at a concentration ratio of 10:1 was significantly higher than at 5:1. **d**. Effect of cinobufagin on the expression of CRF in the supernatant in the co-culture model. Cinobufagin promoted the expression of CRF in the supernatant. When the concentration of cinobufagin was 1/40, the effect was remarkable. Compared with control group, *P < 0.05, **P < 0.01; comparing at different times or different concentration ratios, ^##^*P* < 0.01.

Isolated spleen lymphocytes were suspended in RPMI1640 medium in a humidified incubator with 5% CO_2_ at 37°C. They were round, small, the membrane was integrated and refraction was good. The exclusion staining rate for trypan blue was more than 95%. H22 hepatoma cells were round, and some large and some small, with the different sizes reflecting multiples of splenic lymphocytes; when the culture density was high, cells clustered in the center. In the co-culture model, spleen lymphocytes were closely packed around hepatoma cells. The cells remained in good condition for up to 48 h and then gradually declined (Figure 5a).

In the co-culture model, after 24 h, compared with the control group, it was clear that cinobufagin promoted the release of β-END from immune cells, the differences were statistically significant (*P* < 0.01). After 48 h, cinobufagin still promoted the release of β-END from immune cells, the differences were statistically significant (P <0.01 or P <0.05). The results after 24 h compared to 48 h were statistically significant (*P* < 0.01) (Figure 5b).

Compared with the control group, in the co-culture model when the spleen lymphocyte and H22 hepatoma cell concentration ratio was 10:1 or 5:1, different concentrations of cinobufagin stimulated the immune cells to release β-END, the differences were statistically significant (*P* < 0.01). The expressions of β-END at a concentration ratio of 10:1 was significantly higher than that at 5:1 (*P* < 0.01), except cinobufagin concentration of 1/70 (Figure 5c). Cinobufagin also significantly increased the levels of CRF in the supernatant compared with the control group. When the concentration of cinobufagin was 1/40, the effects were quite remarkable (*P* < 0.01) (Figure 5d).

**Figure 6:**
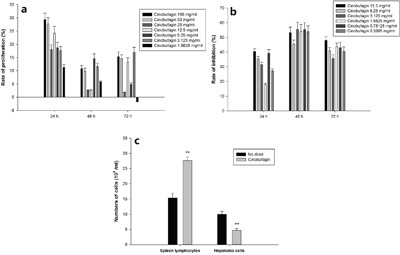
Effect of cinobufagin on spleen lymphocytes and hepatoma cells **a**. cinobufagin promoting spleen lymphocytes proliferation. Cinobufagin obviously promoted separated spleen lymphocyte proliferation as determined by the MTT method, especially after 24 h when promotion was at a peak (~30%). **b**. cinobufagin inhibiting proliferation of hepatoma cells. Cinobufagin inhibited separated hepatoma cell proliferation. The inhibition rate was greatest at 48 h and lowest after 24 h. **c**. Effect of cinobufagin on spleen lymphocytes and hepatoma cells in co-culture. The number of hepatoma cells was significantly reduced in the cinobufagin group, but in contrast, the number of spleen lymphocytes increased significantly. Compared with no dosing group, **P < 0.01.

Cinobufagin to separate spleen lymphocyte proliferation has obvious effect on promoting proliferation, especially in the 24 h when promoting effect is the largest, the highest close to 30%. 48 h proliferative effect is weak, and 72 h proliferation effect is better than 48 h, but a great difference between the concentration (Figure 6a). Cinobufagin could significantly inhibit hepatoma cell proliferation. The rate of inhibition highest at 48 h and lowest at 24 h (Figure 6b). Spleen lymphocytes and hepatoma cells (concentration ratio 5:1) were co-cultured for 24 h, then gradient centrifugation separation and individually counted. The hepatoma cell count was significantly reduced in the cinobufagin group, but their spleen lymphocyte numbers increased significantly, the results had statistically significant difference (Figure 6c) (*P* <0.01). After cinobufagin intervention 24 h, immune cells proliferation rate was 80%, hepatocellular carcinoma cell inhibition rate was 53%.

**Figure 7:**
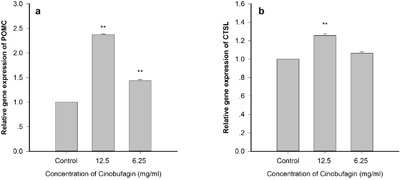
Effect of cinobufagin on immune cell gene expression of POMC and CTSL by RT-PCR Cinobufagin can obviously promote immune cell POMC and CTSL gene expression levels in the co-culture model. When the cinobufagin concentration was 12.5 mg/mL, the effect was best. Compared with control group, *P < 0.05, **P < 0.01.

Real-time PCR demonstrated that cinobufagin obviously promoted immune cell POMC gene expression levels in the *in vitro* co-culture model compared with the control group, and the results were statistically significant (*P* <0.01). When the cinobufagin concentration was 12.5 mg/mL (1/40), the effect was best (Figure 7a). *In vitro* co-culture system, CTSL gene expression levels was also increased, and cinobufagin could promote immune cells CTSL gene expression levels at a certain extent. When the cinobufagin concentration was 12.5 mg/mL, the result was statistically significant (*P* <0.01) (Figure 7b).

**Figure 8:**
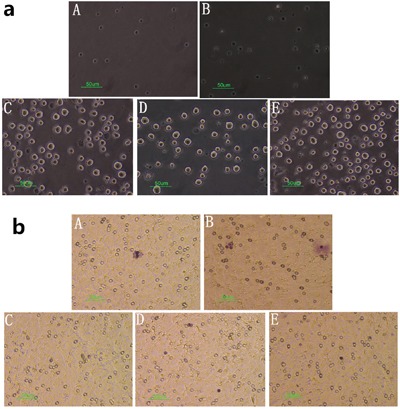
Effect of cinobufagin on spleen lymphocytes in the migration assay (A) Spleen lymphocytes + cinobufagin 1/40, (B) Spleen lymphocytes + cinobufagin 1/320, (C) Spleen lymphocytes + hepatoma cells, (D) Spleen lymphocytes + H_22_ hepatoma cells + cinobufagin 1/320, (E) Spleen lymphocytes + H_22_ hepatoma cells + cinobufagin 1/40. **a**. Cell morphology in the lower layer of the migration assay (40×). Spleen lymphocytes were suspended in the lower layer and therefore could not grow on the opposite side of the polycarbonate membrane. **b**. Cell morphology on the opposite side of the polycarbonate membrane in the migration assay (40×). The pictures show crystal violet staining, purple for spleen lymphocytes. The numbers were lower as they could not grow on the opposite side of the polycarbonate membrane.

The potential effect of cinobufagin on spleen lymphocyte chemotaxis was determined using a migration assay as specified in the methods section. Spleen lymphocytes were added to the upper layer and either hepatoma cells and different concentrations of cinobufagin to the lower layer or just different concentrations of cinobufagin. The results clearly showed that cinobufagin had a chemotaxis on spleen lymphocytes. In co-culture system, we found that cinobufagin at a concentration of 1/320 was the most effective concentration for stimulating chemotaxis when just add cinobufagin group; for hepatoma cells, a concentration of 1/40 cinobufagin was the most effective (Figure 8a, 8b). After calculating the chemotactic index, a value > 2 was shown to produce meaningful results, which were documented in Table 1.

**Table 1:** Number of cells migrating in each group

Groups	N	SD	Chemotactic index
Lymphocytes + H_22_	4.33	0.577	
Lymphocytes + cinobufagin 1/40	12	2	2.771^*^
Lymphocytes + cinobufagin 1/320	31.67	2.517	7.314^*^
Lymphocytes + H_22_ + cinobufagin 1/40	20.33	1.528	4.695^*^
Lymphocytes + H_22_ + cinobufagin 1/320	6.77	2.082	1.564*

Compared with lymphocytes + H_22_ group, *P < 0.05, **P < 0.01.

## DISCUSSION

### Effects of cinobufagin on cancer pain model and co-culture model

In the present study, a pain model was successfully developed by injecting hepatoma cell lines H22 into the right hind paw of Kunming mice [20, 21], with a tumor formation rate of 100%. The mice exhibited pronation and other spontaneous pain behaviors and a decreased flexibility of action. Our research on hind paw cancer pain mice model found that from 0.5 h after initial administration, the thermal and mechanical threshold of model group mice were significantly lower than that of control group mice, which indicated that thermal and mechanical stimulation are two sensitive ways to reflect the pain behavior change of mice treated by different drugs, and could be used as indicators to evaluate the model and drug effects. Following 2, 4, 6 and 8 days of modeling, the thermal stimulation and mechanical pain thresholds in the model group were lower than in the control group and cinobufagin significantly improved the thermal and mechanical pain thresholds. It suggested that cinobufagin and morphine had therapeutic effects on the cancer pain model mice.

Co-culture of two or more types of cells perhaps better reflects the environment *in vivo* [22–23]. Because of the mutual influence between cells, cell characteristics such as proliferation and differentiation play important roles in the regulation of development. The co-culture system can place a variety of cells in the same environment (e.g. endothelial cells and smooth muscle cells, tumor cells and endothelial cells, hepatocytes, fibroblasts and epithelial cells and corneal keratocytes and so on), to simulate the interaction between them. We use this system to study the complex interaction between cells [24–27].

In the present study, splenic lymphocytes and H22 hepatoma cells were cultured *in vitro* using a direct co-culture system which can make the two cells make adequate contact with each other and is closer to the body tissue environment. Spleen lymphocytes have a role in the killing H22 hepatoma cells and the latter have a erosion effect on spleen lymphocytes, hence the two co-cultured cells need to be cultured in the right proportion. We concluded that spleen lymphocytes and H22 hepatoma cells can be co-cultured at a proportion between 5:1 and 10:1 respectively, but that a ratio of 10:1 is the best combination. The cinobufagin concentration range should be carefully controlled because if the concentration is too high the liver cancer cells will die and the experimental observations will necessarily cease. In contrast, if the concentration is too low will not have an effect on cancer pain. Moreover, the time that spleen lymphocytes and H22 hepatoma cells are *in vitro* cell co-culture is one week at the most, so the best time they can be in a co-culture system is just four or five days. In short, the many features of these two cell types are very similar and our study successfully established an *in vitro* co-culture model which has laid the foundations for a further study of the fundamental mechanisms involved.

### Peripheral immunological analgesia mechanisms

Cinobufagin has been reported to possess various pharmacological properties, including immunoregulatory and anticancer effects [28–31]. It has also been used as an effective traditional Chinese medicine to treat conditions like swelling, pain and heart failure [32]. The present study was designed to investigate the effects of cinobufagin on cancer pain and its peripheral immunological analgesia mechanisms. A cancer pain model and cell co-culture *in vitro* was studied from three main perspectives, namely immunological and analgesic properties and tumor suppression. It was confirmed that cinobufagin can inhibit the growth of tumor cells, promote the proliferation of immune cells and increase the secretion of the analgesic factor β-END by stimulating its synthesis and release. The primary focus of the research was to establish whether cinobufagin could induce peripheral analgesia. Morphine increased the protein expression of μ-OR in the tumor and adjacent tissues and β-END in the plasma, and the local analgesic factors of β-END and its precursor POMC did not change significantly; while the expressions of POMC, β-END and μ-OR in cinobufagin treated mice were significantly higher than in model group mice. It is certainly true that the analgesic mechanisms evoked by morphine differ from those of cinobufagin, which is an analgesic mechanism in the periphery rather than in the central nervous system.

Opioid peptide family includes enkephalin (ENK), endorphins (EP), dynorphin (Dyn) and later discovered orphanin FQ, endomorphins, etc [33]. Endogenous opioid peptides (EOP) are opioid active substances synthesized in mammals naturally, and exogenous β-END intravenous injection cannot alleviate the inflammatory pain of mice paw induced by stress, and somebody put up that opioid peptides may be released from local tissue of inflammatory claw [34]. Further research detected a considerable amount of β-END and encephalin and the mRNA of POMC (the precursor of β-END) from various immune cells of inflammatory tissue such as T lymphocytes, B lymphocytes, monocytes and macrophages [35], moreover, the content of β-END in the inflammatory tissue was positively correlated with the number of immune cells [36–37]. Immune cells in inflammatory tissue synthesis a large number of POMC, which is enzymatically processed to form a series of peptide hormones substances containing α-MSH, ACTH and β-END, etc. β-END is stimulated by corticotropin-releasing factor (CRF) and interleukin-1 (IL-1) to release into the extracellular, and conjunction with nerve endings of opioid receptors of the local inflammation tissue to play an endogenous analgesia.

The occurrence and development of tumors are closely linked with inflammatory tumor infiltrating lymphocytes (TILs), mainly CD3^+^ and CD8^+^ T-cells among others. Cinobufagin has been shown to enhance T-cell immune functions [38], enhance the secretion of IL-2 and IL-10 and increase the phagocytosis ability of macrophages [39]. This drug also reduces the incidence of infection in patients with cancer [40], raises the ratio of CD3^+^/CD8^+^ and CD3^+^/CD56^+^ cells and improves the cytotoxic activity and cell killing activity of cytokine-induced killer (CIK) cells [41]. Compared with the model group, the protein expression of CD3^+^ and CD8^+^ in the tumor and adjacent tissues of cinobufagin treated mice was significantly increased, suggesting that cinobufagin has a promoting effect on TILs.

When a tumor occurs, the body's stress response can trigger analgesia by releasing central opioid peptides in the early stages and subsequently by peripheral opioid peptides release [42]. *In vitro* cell experiments show that immune cells and tumor cells co-culture secrete the highest levels of β-END when the stimulus conditions for 24 h. The results further demonstrate that cinobufagin has a positive effect on the proliferation of immune cells (up to 30%), and that the highest proliferation rate occurred at 24 h. By promoting immune cell proliferation, cinobufagin enhances immunity and improves the survival time of cancer patients. The increase in immune cell numbers and obviously promoting immune cell POMC and CTSL gene expression levels, leading to the synthesis of more β-END, which has an indirect analgesic effect. Cinobufagin also had a positive effect on immune cell proliferation in the co-culture system (liver cancer cells and immune cells), which was much stronger than with a single type of immune cell, that explain cinobufacini can promote tumor cells induced immune cells proliferation. Cinobufagin can inhibit cancer cell proliferation, and its this effect is likely to be be achieved by enhancing immunity, that is, to induce TILs proliferation. Although, the inhibition rate of tumor cells by cinobufagin was most effective within 48 h, the analgesic action of cinobufagin and its effect on immune cells proliferation reached a peak within 24 h. When cinobufagin inhibited tumor cells, 48h tumor cells at least. At the same time, the stimulatory action of cinobufagin by tumor cells on immune cells become weaker. It can be concluded that the function of immune cells could play a deeper role in the cancer if stimulated sufficiently. Moreover, cinobufagin has a chemotactic effect on immune cells, and also has a chemotactic effect on immune cells induced by stimulation of liver cancer cells. Thus, cinobufagin can recruit immune cells to the injured tissue and thus produce useful defense and analgesic actions.

## MATERIALS AND METHODS

### Animals

Female Kunming mice (Mus musculus) (20 ± 2g) were purchased from the Laboratory Animal Institute of Hubei Disease Control Center (Wuhan, China). Mice were housed under constant conditions at 22 ± 2°C, 60 ± 5% humidity and on a 12 h light-dark cycle. They were fed ad libitum and acclimatized in a non-stressful environment for at least 1 week prior to experiments. Experiments were performed in accordance with the Guide for the Care and Use of Laboratory Animals of China Three Gorges University, and approved by the Ethical Committee of Animal Experimentation. The whole laboratory procedure was carried out with the permission and under the surveillance of the ethics committee.

### Preparation of H22 hepatoma cells

H22 murine hepatoma cell line was purchased from the Shanghai Institute of Materia Medica, Chinese Academy of Sciences, and were cultured by the Immune Research Center at our university. And it was serially cultured in Kunming mice for three generations, and ascites were extracted after 7 days. The sample was washed in D-Hanks solution and centrifuged twice at 800 r/min × 5min and dyed with trypan blue to detect whether the survival rate ≥95%. The cell suspension was adjusted to 6 × 10^7^/mL, which was placed in ice until use.

### Establishment of the hind paw cancer pain model, grouping and administration

Mice in the model groups were injected subcutaneously with 0.1 mL 6×10^7^ of H22 hepatoma cells, and mice in the control group were injected with 0.1 mL of normal saline (NS). The whole procedure was carried out aseptically and was completed within 1 h. 36 mice of 48 female Kunming mice, which were administrated tumor cells, were randomly divided into 3 groups: model group, morphine group (morphine, 8mg/kg/day, i.p.), and cinobufagin group (cinobufagin injection, 2.5 g/kg/day, i.p., Jinchan Biochemical Co. Ltd. Anhui, China), with 12 individuals in one group; the remaining 12 normal mice which were not administrated tumor cells were selected control group. The experiment mice were administrated homologous drug, respectively; mice in the control and model groups were administered NS, respectively, once daily lasting for 8 days.

### Measurement of thermal hyperalgesia and mechanical hyperalgesia

The pain behavior was recorded before treatment and 0.5, 1.0, 1.5, 3 and 6 h after the initial administration and then on the 2th, 4th, 6th and 8th day. Thermal hyperalgesia [43] was measured using a radiant heat pain measurement instrument (Chengdu Taimeng, China) in a quiet environment (room temperature 22 ± 2°C). The mice were placed in a plexiglass cage and the experiment was performed using an intense light beam to irradiate the center skin of the right hind paw after the mice were adapted to the quiet environment. The time taken for mice to draw back their paw was recorded. The duration of the radiant heat intensity was set to 5-15 seconds for the normal mouse paw withdrawal latency (PWL). Each mouse was tested 3 times, the interval between each was 10 minutes, and the average value was calculated. An upper limit of 20 seconds was set as the PWL to prevent burns. Mechanical hyperalgesia [44] was measured by IITC von Frey 2390 (Woodland Hills, USA) in a quiet environment (room temperature 22 ± 1°C). The mice were placed on a special glass grid, adapted for the quiet environment, and the experiments were performed. Briefly, the center skin of the right hind paw was stimulated and the PWL was determined. Each mouse was tested 3 times, with a 10 minutes interval between each measurement and the average value was calculated.

### Analysis of β-END, CRF, and IL-1β in the plasma and tumor tissues homogenate by ELISA

Blood was harvested from the eyeballs of 6 mice from each group and immediately placed in clean eppendorf (EP) tubes containing heparin, centrifuged for 10 min at 4°C, and the supernatant plasma transferred to clean EP tubes and stored at −80°C for analysis. The right hind paw was depilated using 8% sodium sulfide solution (pH=11.0) and the tumor tissues obtained by deboning. A weight/volume ratio of 1:9 plus NS which was 10% of the homogenate (at low temperature), was centrifuged (3,000 rpm/min) for 15 minutes at 4°C, and the supernatant plasma transferred to clean EP tubes and stored at -80°C for analysis. Samples were analyzed immediately according to the manufacturer's protocols (BOSTER, Wuhan, China). The samples were examined at an absorbance at 450 nm in an enzyme-linked immunosorbent assay (ELISA) plate reader (BIO-RAD, USA).

### Immunohistochemistry analysis of β-END, POMC and μ-OR; immunofluorescence analysis of CD3^+^, CD4^+^ and CD8^+^

The remaining six mice in each group were sacrificed to obtain the right hind paw. The paw was depilated using 8% sodium sulfide solution, fixed in 10% formaldehyde solution for 24 h and decalcificed by 30% acetic acid solution for 10 days. The tissue was then placed in 70% ethanol solution and embedded in paraffin for sectioning. A streptomycin-avidin–peroxidase assay kit was used for immunohistochemistry. Positive results were observed under an optical microscopy when hyalomitome and the cell membrane appeared brown (β-END, POMC and μ-OR) in tumor tissues. Immunofluorescence slices were prepared as for the immunohistochemistry slices (vide supra). Positive results for CD3^+^, CD4^+^ and CD8^+^ were observed using optical microscopy in tumor and adjacent tissues.

### Mice spleen lymphocyte and H22 hepatoma cell co-culture

The H22 hepatoma cell line was serially cultured in Kunming mice for three generations and 0.1 mL ascites were extracted after 7 days and added to 5 mL of PBS. The sample was centrifuged twice at 800 rpm/min for 5 minutes and 5 mL of RPMI1640 medium (GIBICO, USA) added. The cell concentration was adjusted to 2~3×10^5^/mL. In euthanized Kunming female mice (25-30 g), the spleen was stripped aseptically and washed twice in PBS and then added to 5 mL of mice lymphocyte separation medium (Blood Institute, Chinese Academy of Medical Sciences, Tianjin, China). The spleen was squeezed through an 80 mesh screen, and the separated liquid was filtered through a 200 mesh screen. 1 mL of RPMI1640 medium was added to second separated liquid which was then centrifuged at 1,500 rpm/min for 15 minutes, when then the liquid precipatated into 4 distinct layers. The second layer was the white lymphocytes which were washed with 5 mL PBS, centrifuged at 1500 rpm/min for 15 minutes. The sample was then washed twice with PBS and 5 mL of RPMI1640 medium added. The cell concentration was adjusted to 1~2×10^6^/mL.

Splenic lymphocytes and H22 hepatoma cells were cultured *in vitro* using a direct co-culture system. First, the cell culture medium of the two cell types was supplemented by added calf serum 1640 complete medium. Second, these two cell types are suspended cells, which are difficult to co-culture and are not easy to separate. We used different centrifugal forces to carry out a gradient centrifugation. After a 600-1000 rpm for 3 min, H22 hepatoma cells were precipitated and the supernatant transferred to another receptacle. Then, a further centrifugation at 1500 rpm for 8-10 min precipitated the spleen lymphocytes. Under the microscope, the two types of cells exhibited completely different profiles. The volume of splenocytes was very small which had an appearance of cylindrical granular shapes with even scattering. In contrast, the H22 hepatoma cell volume was several times larger than the splenocytes, which were round, large and small with some clustered together allowing for easy identification of each cell type.

### Different concentrations of cinobufagin promote lymphocytes to release β-END in co-culture model at different time

H22 hepatoma cells and spleen lymphocytes were prepared according to the method described above. A concentration of 2 × 10^5^/mL and 1 × 10^6^/mL respectively (concentration ratio approximately 1:5) was added to 24-well culture plates (500 μL in each well), mixed and placed in a humidified incubator with 5% CO_2_ at 37 °C. After 4 h, cinobufagin injection (0.5 g/mL), which was serially diluted by 1/5, 1/10, 1/20, 1/40, 1/80 and 1/160, was injected (500 μL). Each concentration was tested in triplicate. Supernatants (500 μL) were collected after 24 h and 48 h and centrifuged at 3,000 rpm/min for 5 minutes. Aliquots of 50 μL of the supernatants were immediately analyzed according to the manufacturer's protocols.

### Different concentrations of cinobufagin promote lymphocytes to release β-END in co-culture supernatants at different proportion

H22 hepatoma cells and spleen lymphocytes were prepared at concentrations of 2 × 10^5^/mL, 1 × 10^6^/mL (1:5); 2 × 10^5^/ mL, 2 × 10^6^/mL (1:10) and added to a 24-well culture plate (500 μL in each well), mixed and placed in an incubator containing 5% CO_2_ at 37 °C. After 4 h, cinobufagin injection (0.5 g/mL), which was serially diluted by 1/20, 1/30, 1/40, 1/50, 1/60, 1/70, 1/80, 1/160 and 1/320, was injected (500 μL). Each concentration was studied in triplicate. Supernatants (500 μL) were collected after 24 h, centrifuged at 3,000 rpm/min for 5 minutes and 50 μL aliquots analyzed immediately according to the manufacturer's protocols.

### Effect of different concentrations of cinobufagin on the expression of CRF in co-culture supernatants

H22 hepatoma cells and spleen lymphocytes were prepared at concentrations of 2 × 10^5^/mL and 2 × 10^6^/mL respectively (1:10 concentration ratio) and added to 24-well culture plates (500 μL in each well), mixed and placed in an incubator containing 5% CO_2_ at 37 °C. After 4 h, cinobufagin injection (0.5 g/mL), which was diluted by 1/30, 1/40, 1/50, 1/60 and 1/70, was injected (500 μL). Each concentration was analysed in triplicate. Supernatants (500 μL) were collected after 24 h, and then centrifuged at 3,000 rpm/min for 5 minutes and 50 μL aliquots analyzed immediately according to the manufacturer's protocols.

### Effect of different concentrations of cinobufagin on the proliferation of spleen lymphocytes and H22 hepatoma cells

Spleen lymphocytes (5 × 10^5^/mL) was prepared, and added to a 96-well culture plate (100 μL in each well). 150 μL of PBS was added around the wells and the plate placed in an incubator containing 5% CO_2_ at 37 °C. After 4 h, cinobufagin injection (0.5 g/mL), diluted by 1/5, 1/10, 1/20, 1/40, 1/80, 1/160 and 1/320, was injected (50 μL). Each concentration was tested 5 times. The cells were cultured for 24 h, 48 h and 72 h. For analysis before 4 h, 20 μL MTT (5 mg/mL, AMRESCO, USA) was added to each well, then MTT solution was removed gently and 150 μL of dimethyl sulfoxide was added to each well for 15 min incubation. The absorbance of each sample was measured at 570 nm. Calculate the proliferation rate according to the following formula: rate of proliferation = (treatment group - control group) / control group.

H22 hepatoma cells (5 × 10^4^/mL) was prepared, and added to a 96-well culture plate (100 μL in each well). 150 μL of PBS was added around the wells and the plate placed in an incubator containing 5% CO_2_ at 37 °C. After 4 h, cinobufagin injection (0.5 g/mL), diluted by 1/40, 1/80, 1/160, 1/320, 1/640 and 1/1280, was injected (50 μL). Each concentration was tested 5 times. The cells were cultured for 24 h, 48 h and 72 h. For analysis before 4 h, 20 μL MTT (5 mg/mL, AMRESCO, USA) was added to each well, then MTT solution was removed gently and 150 μL of dimethyl sulfoxide was added to each well for 15 min incubation. The absorbance of each sample was measured at 570 nm. Calculate the inhibition rate according to the following formula: rate of inhibition = (control group - treatment group) / control group.

### Effect of cinobufagin on the proliferation of spleen lymphocytes after co-culture

H22 hepatoma cells (1 × 10^5^/mL) and spleen lymphocytes (1 × 10^6^/mL) were prepared, seeded in 9 mm culture dishes (6 dishes), with 2 mL in each dish. Cinobufagin injection (0.5 g/mL) was diluted 1/40, and added to three of the dishes (1 mL; 3 dishes contained no drugs) and the dishes placed in an incubator containing 5% CO_2_ at 37 °C. After 24 h, samples were centrifuged at 1000 rpm/min for 5 minutes (Gradient centrifugation for two kinds of cell separation, H22 hepatoma cells precipitated at this time), and then the supernatants were further centrifuged at 1500 rpm/min for 10 minutes (when spleen lymphocytes precipitated). RPMI1640 medium were used for the two cells suspension and the cells were counted compared with no inoculation, and the proliferation and inhibition rate were calculated.

### Quantitative real-time PCR

H22 hepatoma cells (1×10^5^/mL) and spleen lymphocytes (1×10^6^/mL) were prepared and added to a 6-well culture plate (1 mL in each well). Cinobufagin injection (0.5 g/mL) was diluted 1/40 or 1/80 and added to four of wells (1 mL; 2 wells contained no drugs) and placed in an incubator containing 5% CO_2_ at 37°C. After 24 h, samples were centrifuged as described above (vide supra). Total RNA was isolated from the samples using TRIzol® reagent (Invitrogen, USA) following the manufacturer's protocol. Briefly, after chloroform extraction, RNA was precipitated with isopropanol and the pellet washed twice in 75% ethanol. After air-drying, the RNA was resuspended in DEPC treated water. Both the quantity and the quality of the total RNA were examined by gel electrophoresis and by UV spectrophotometry. Complementary DNA was synthesized using reverse-transcriptase (Promega, China). In this study, β-actin was used as the reference gene to normalize expression levels. Primers for qRT-PCR were designed using Primer Express software version 2.0 (Applied Biosystems, Inc.). POMC forward primer, 5’-GTG TGG GGA GAT GGC AGT CCAG-3’; POMC reverse primer, 5’-CAC CGT AAC GCT TGT CCT TGGG-3’; CTSL forward primer, 5’-TTA CTC CTT TTG GCT GTC CTCT-3’; CTSL reverse primer, 5’-ATA TCG CTC TCC TCC ACT CTTC-3’; β-actin forward primer, 5’- CTG AGA GGG AAA TCG TGCGT -3’; β-actin reverse primer, 5’- CCA CAG GAT TCC ATA CCC AAGA -3’.

qRT-PCR was performed using an ABI 7500 real-time PCR system (Applied Biosystems, Inc.). The reactions were set up in 20 μL volumes containing 2× SYBR Green qPCR Mix. The relative gene expression level was calculated from the target and β-actin using the following formula: mRNA relative expression = 2 - (Ct of target - Ct of β-actin).

### Migration assay

H22 hepatoma cells (5×10^4^/mL) and spleen lymphocytes (1×10^5^/mL) were prepared by the method described above (vide supra). Transwell filters were added to 24-well plate according to the manufacturer's instructions (Costar, USA). Spleen lymphocytes (200 μL) were added to the upper layer. Cinobufagin injection (0.5 g/mL) was diluted 1/40, 1/320 and 1/1280. Six groups of samples were added to the lower layer: H22 hepatoma cells (600 μL); H22 hepatoma cells (500 μL) + cinobufagin (100 μL, 1/40); H22 hepatoma cells (500 μL) + cinobufagin (100 μL, 1/320); H22 hepatoma cells (500 μL) + cinobufagin (100 μL, 1/1280); cinobufagin (600 μL, 1/40); cinobufagin (600 μL, 1/320). The 24-well plate was placed in an incubator containing 5% CO_2_ at 37°C. After 24 h, a cotton swab was used to wipe the cells in front of polycarbonate membrane, which were fixed paraformaldehyde and stained with crystal violet to visualise cell membranes. Spleen lymphocytes in the lower layer were then counted (four horizons). Drug chemotactic activity is often expressed in chemotactic index (CI), CI = the number of cells in the experimental group / the number of cells in the control group.

### Statistical analysis

All quantitative data derived from this study were analyzed statistically. The results were expressed as means ± standard deviation (SD). A database was set up using SPSS ver. 19.0 software (SPSS Inc., Chicago, IL). Differences among groups were analyzed by one-way analysis of variance (ANOVA) or repeated measures analysis of variance. If the variance was regular, a Dunnett test was used and if the variance was irregular the Tamhane's T2 method was used. In the migration assay, an independent sample t-test was used for data analysis. *P* value < 0.05 was taken as the level of statistically signifcant.

## CONCLUSIONS

Cinobufagin can relieve cancer pain in mice and raise the pain threshold, a mechanism likely involving the upregulation of the expression of β-END in the tumor tissue and also the proliferation of TILs. Cinobufagin significantly improved β-END synthesis by increasing the expression of POMC, CTSL and stimulated the release of β-END by raising the levels of inflammatory chemokines in the tumor and adjacent tissues. As a result, the cinobufagin stimulated increase in β-END binding to the μ-opioid receptor will play an important role in peripheral analgesia. In addition, cinobufagin not only inhabits cancer cells but also promotes immune cells hyperplasia providing a more effective immune defense system. Cinobufagin may exert its local peripheral analgesic effect by activating the POMC/β-END/μ-OR pathway by promoting proliferation of immune cells, recruitment and therefore increase β-END levels.
